# Effects of Oral Gamma-Aminobutyric Acid (GABA) Administration on Stress and Sleep in Humans: A Systematic Review

**DOI:** 10.3389/fnins.2020.00923

**Published:** 2020-09-17

**Authors:** Piril Hepsomali, John A. Groeger, Jun Nishihira, Andrew Scholey

**Affiliations:** ^1^Unilever R&D, Bedford, United Kingdom; ^2^Department of Psychology, School of Social Sciences, Nottingham Trent University, Nottingham, United Kingdom; ^3^Department of Medical Management and Informatics, Hokkaido Information University, Hokkaido, Japan; ^4^Centre for Human Psychopharmacology, School of Health Sciences, Swinburne University, Hawthorn, VIC, Australia

**Keywords:** gamma amino butyric acid (GABA), stress, sleep, nutrients, food supplements

## Abstract

Gamma-aminobutyric acid (GABA) is a non-proteinogenic amino acid and is the main inhibitory neurotransmitter in the mammalian brain. GABA's stress-reducing, and sleep enhancing effects have been established. However, although several human clinical trials have been conducted, results regarding the role of natural and/or biosynthetic oral GABA intake on stress and sleep are mixed. We performed a systematic review to examine whether natural and/or biosynthetic oral GABA intake has an effect on stress and sleep. We systematically searched on PubMed database for studies published up to February 2020 following PRISMA guidelines. Only placebo-controlled human trials that assessed stress, sleep, and related psychophysiological outcomes as a response to natural GABA (i.e., GABA that is present naturally in foods) or biosynthetic GABA (i.e., GABA that is produced via fermentation) intake were included. Fourteen studies met the criteria and were included in the systematic review. Although more studies are needed before any inferences can be made about the efficacy of oral GABA consumption on stress and sleep, results show that there is limited evidence for stress and very limited evidence for sleep benefits of oral GABA intake.

## Introduction

Gamma-aminobutyric acid (GABA) is a four-carbon non-proteinogenic amino acid that is present in bacteria, plants, and vertebrates. Initially, it was discovered in plants (Steward et al., 1949), it was then identified in the mammalian brain (Roberts and Frankel, 1950), and subsequently in animals (Roberts and Eidelberg, 1960) and several other organisms—including bacteria and fungi (Bouche et al., 2003). In vertebrates, it is generated by the irreversible α-decarboxylation reaction of L-glutamic acid or its salts, catalyzed by glutamic acid decarboxylase enzyme (Satya Narayan and Nair, 1990) and functions as an inhibitory neurotransmitter in the central nervous system (CNS) (Roberts and Frankel, 1950; Petroff, 2002), It has also been found in several peripheral tissues (Erdö, 1985). GABA is critical to the functioning of the CNS, where ~60–75% of all synapses are GABAergic (Schwartz, 1988).

In addition to its role as a neurotransmitter, GABA also exists naturally in various foods, such as tea, tomato, soybean, germinated rice, and some fermented foods, and could be obtained from a normal diet (Diana et al., 2014; Rashmi et al., 2018). For example, white tea and adzuki beans contain 0.5 and 2.01 g/kg GABA, respectively (Zhao et al., 2011; Liao et al., 2013). On the other hand, much higher concentrations of GABA could be produced by lactic acid bacteria (LAB) fermentation (Dhakal et al., 2012). For instance, by using *Lactobacillus brevis* NCL912 strain, 103.5 g/l GABA could be produced (Li et al., 2010). Recently, LAB GABA has gained significant attention and has been widely used as a functional food ingredient in various markets due to its potential health benefits associated with GABA (Boonstra et al., 2015).

It is worth mentioning that GABA has long been thought to be unable to cross the blood–brain barrier (BBB) (Kuriyama and Sze, 1971; Roberts, 1974), which raises questions about the mechanisms of action behind its health benefits. However, there are various accounts regarding GABA's BBB permeability. While some researchers argue that only small amounts of GABA cross the BBB (Knudsen et al., 1988; Bassett et al., 1990), with the discovery of GABA-transporter systems in the brain (i.e., passing of solutes by transcytosis, carrier-mediated transport, or simple diffusion of hydrophobic substances), others believe that the substantial amounts of GABA could cross the BBB (Takanaga et al., 2001; Al-Sarraf, 2002; Shyamaladevi et al., 2002). Additionally, as GABA is also present in the enteric nervous system, it has been considered that GABA may act on the peripheral nervous system through the gut-brain axis (Cryan and Dinan, 2012). Although there is some evidence showing that biosynthetic GABA could reach the human brain as evidenced by various EEG responses (Abdou et al., 2006; Yoto et al., 2012), to date, there are no data showing GABA's BBB permeability in humans. Although it has been shown that the blood GABA levels were elevated 30 min after oral GABA intake (Yamatsu et al., 2016), it's not known if oral GABA intake would increase brain GABA concentrations or not.

Given the ubiquitous role of GABA as an inhibitory neurotransmitter, along with its widespread distribution, it is unsurprising that it has been implicated in a large range of behaviors (Olney, 1990). These include anxiety and stress regulation, circadian rhythm and sleep regulation, memory enhancement, mood, and even perception of pain (Diana et al., 2014; Rashmi et al., 2018). Low levels of GABA or impaired GABA functioning is associated with the etiology and maintenance of acute and chronic stress (Jie et al., 2018), anxiety disorders (Nemeroff, 2003) and sleep disturbances such as insomnia (Gottesmann, 2002). Specifically, GABAergic neurons and neurotransmitters regulate the brain circuits in (i) the amygdala to modulate stress and anxiety responses both in the normal and pathological conditions (Nuss, 2015), (ii) cortico-medullary pathways to modulate both rapid eye movement (REM) and Non-REM, particularly slow wave sleep (SWS) sleep (Luppi et al., 2017), and (iii) the suprachiasmatic nuclei (SCN) to modulate circadian rhythm (DeWoskin et al., 2015). Also, allosteric sites on the GABAa receptors allow the level of inhibition of neurons in the relevant brain regions to be regulated with high accuracy, and these sites are the molecular targets of both anxiolytic and hypnotic drugs (Nuss, 2015; Riemann et al., 2015). Hence, the pharmacological treatment of anxiety disorders and insomnia usually employs a benzodiazepine receptor agonist that affects GABAergic transmission (Nemeroff, 2003; Riemann et al., 2015) which act by increasing the binding of GABA to GABAa receptors in order to enhance inhibitory signals to cell groups regulating arousal. This results in reduced stress and anxiety, decreased sleep latency, and increased sleep continuity (Gottesmann, 2002; Nemeroff, 2003; Nuss, 2015).

Whilst a limited number of human trials with a wide range of methods (in terms of the dose of GABA, duration of the intervention, and measures used to assess stress and sleep) have investigated the impact of non-pharmacological approaches to reduce stress and improve various aspects of sleep by employing natural and biosynthetic GABA intake, to our knowledge, this area of research has not been reviewed systematically. Despite the high methodological variability of the studies included in the current review, the objective of this review is to carry out a systematic review and assess the robustness of scientific evidence supporting the beneficial effects of oral GABA (natural or biosynthetic) intake on stress, sleep, and related psychophysiological measures.

## Methods

### Selection of Studies

#### Inclusion Criteria

The inclusion criteria were the following:

- Outcome measures: stress, anxiety, sleep and/or related psychophysiological parameters
- Design: randomized controlled trials and quasi-experimental trials
- Participants: Any age or gender, healthy or unhealthy participants.

#### Exclusion Criteria

The exclusion criteria were the following:

- Product: Synthetic GABA (i.e., Pharmaceutical-grade substances)
- Design: Case report, letter to editor, conference paper, thesis, personal opinion, or commentary
- Animal studies, *in vitro* and *ex vivo* studies.

### Data Sources and Search Strategy

We carried out an electronic literature search on PubMed to identify relevant studies. The search was conducted until the beginning of February 2020. The search strings used in search were GABA AND (stress OR sleep) NOT (gabapentin OR pregabalin). Articles were selected according to the Preferred Reporting Items for Systematic Reviews and Meta-Analyses (PRISMA) diagram (Moher et al., 2015; Shamseer et al., 2015). One reviewer (PH) independently selected papers according to the aforementioned inclusion and exclusion criteria. The following information was extracted from all publications:

- *Publication details*: authors, year, journal
- *Participant characteristics*: number of participants recruited, number of participants included in the study, number of participants (intervention), number of participants (control), number of participants (other intervention), health status, gender, and age range
- *Study design*: design and blinding
- *Intervention characteristics*: intervention duration, washout period, GABA format, GABA type (natural or biosynthetic), GABA dose, other intervention types and doses
- *Control characteristics*: presence/absence of control/placebo, control/placebo doses
- *Outcome measures*: stress and sleep questionnaires, cortisol, chromogranin A (CgA), immunoglobulin A (IgA), adrenocorticotropic hormone (ACTH), adiponectin, heart rate and heart rate variability, blood pressure, EEG variables
- *Remarks*: notes on the factors that might affect results/data quality.

Study quality was also assessed by using Cochrane Collaboration's tool for assessing risk of bias in randomized trials (Higgins et al., 2011).

## Results

**Figure 1:**
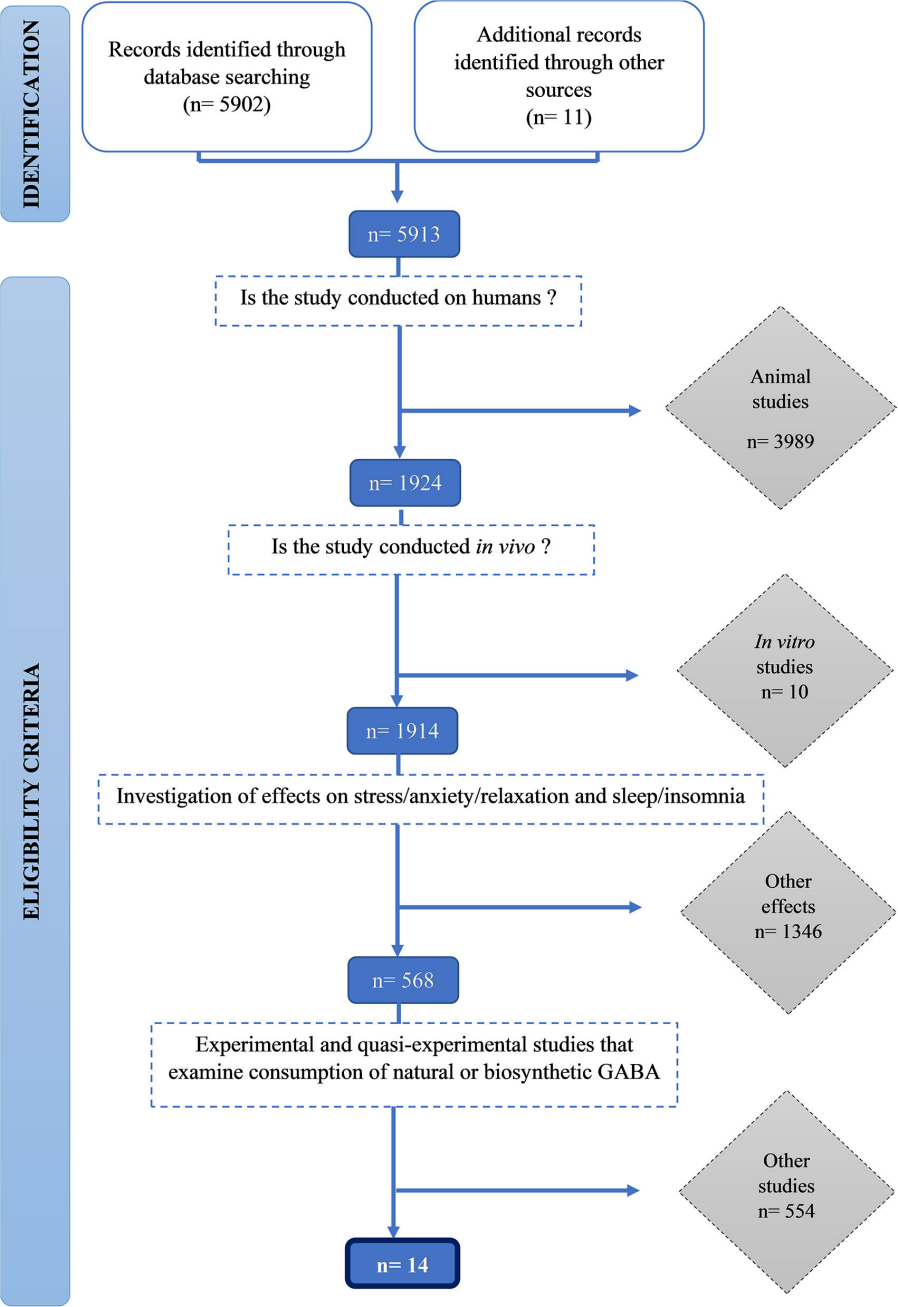
PRISMA flowchart of the selection procedure.

We identified 5,912 publications and screened them for eligibility using inclusion and exclusion criteria. Initially, 3,989 animal studies, then a further 10 *in vitro* human studies were excluded. One thousand three hundred forty-six studies that did not measure stress and sleep-related outcomes were excluded. Finally, 554 studies which did not examine consumption of natural or biosynthetic GABA were excluded. Fourteen studies met all the inclusion criteria were included in this review (Figure 1).

**Figure 2:**
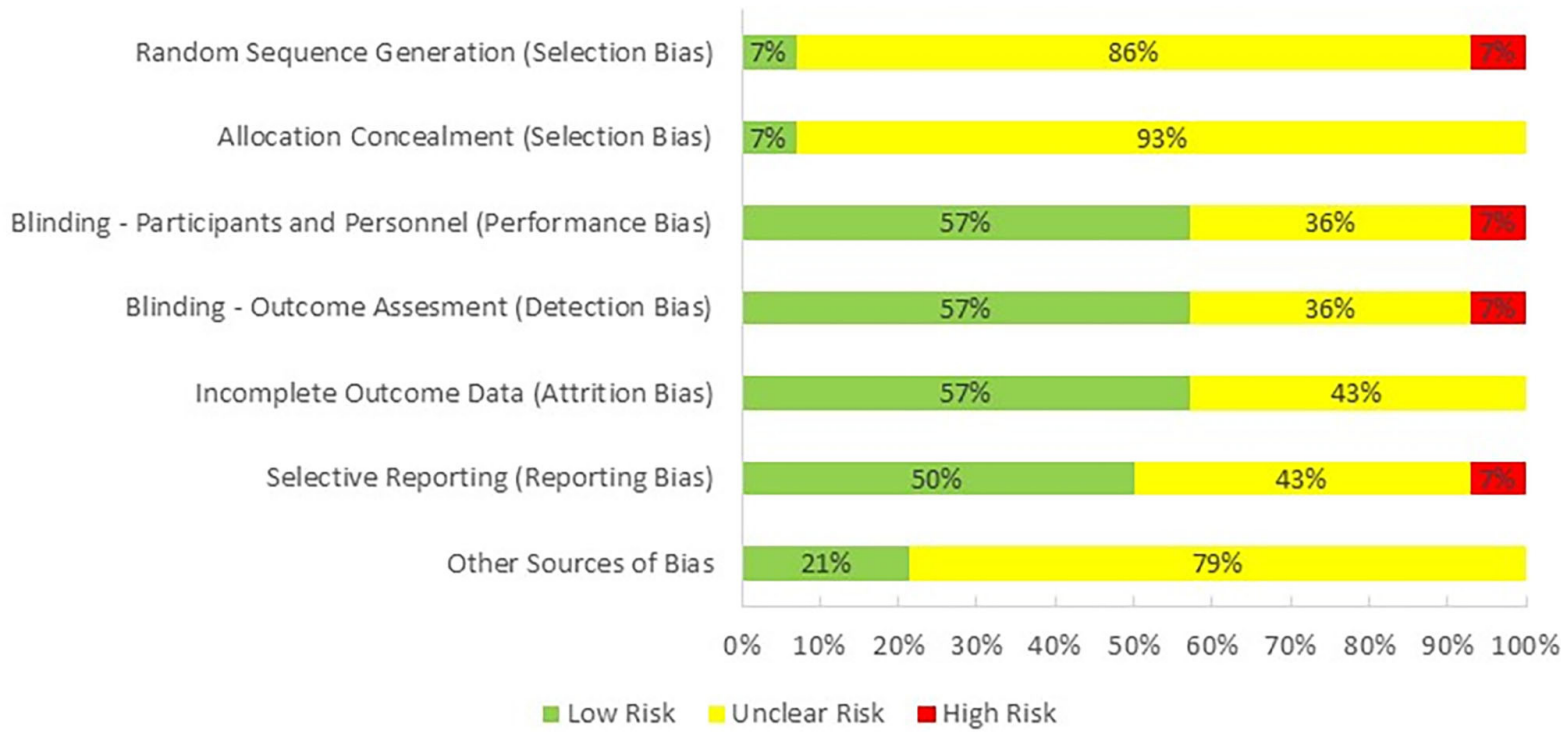
Risk of bias across studies.

One reviewer (PH) evaluated the quality of the studies included in this review by using Cochrane Collaboration's tool for assessing risk of bias in randomized trials (Higgins et al., 2011; Figure 2). The majority of the studies were categorized as having an unclear risk of selection bias, because only one of them reported the method used for random sequence generation and allocation concealment. Performance bias, detection bias, and attrition were observed as having low risk as most of the studies were double-blind and reported all of the outcomes. Risk for reporting bias was unclear as we were not sure if researchers analyzed and reported all of the outcomes that could be extracted from their selected methodologies. Finally, there was an unclear bias for potential conflict of interest as one or more than one authors of 11 studies were employed by an industrial company at the time of publication.

Summaries of all the studies are presented in Table 1 (Methods) and Table 2 (Outcomes).

**Table 1:** Summary of the studies–methodology.

**References**	**Participants (*N* and characteristics)**	**Intervention (I) vs. control (C)**	**Duration of intervention**	**Design**	**Dependent measures**	**Time measures taken**
Hinton et al. (2019)	30 (11 males, age range: 18–30, healthy)	I: 2.01 mg GABA in 200 ml GABA Oolong Tea C: 0.25 mg GABA in 200 ml Oolong Tea	Single dose	Single-blind, controlled, parallel	Immediate stress questionnaire, HRV (TP, LF, HF, LF/HF, RR interval)	Pre-ingestion and 30 min post-ingestion
Yoshida et al. (2015)	39 (19 males, age range: 45–60, healthy)	I: 16.8 mg GABA in 150 g GABA rice/day C: 4.1 mg GABA in 150 g white rice/day	8 weeks	Double-blind, controlled, parallel	VAS for calmness, worry, sleepiness, and feeling of awakening, cortisol, ACTH, adiponectin	Pre-intervention (Week 0) and Weeks 4, 8, and 10
Okada et al. (2000)	20 (females only, age range: 38–52, healthy—post-menopausal)	I: 26.4 mg GABA in rice−3 times a day C: Not clear	8 weeks	Double-blind, controlled, crossover	Kupperman menopause index items	Pre-intervention (Week 0) and Weeks 4, 8, and 16
Fujibayashi et al. (2008)	12 (males only, age range: 21–23, healthy)	I: 30 mg GABA + cellulose (capsule) C: Cellulose (capsule)	Single dose	Double-blind, controlled, crossover	HRV (TP, HF, LF)	Pre-ingestion, 30 and 60 min post-ingestion
Yamatsu et al. (2015)	19 (males only, age range: 24–45, healthy)	I: 20 mg GABA + 280 ml coffee I: 280 ml coffee C: 280 ml water	Single dose^*^	Double-blind, controlled, crossover	CgA	Pre-ingestion, 30 and 60 min post-ingestion
Kanehira et al. (2011)	30 (16 males, age range: 24–43, healthy with chronic fatigue)	I: 25 mg GABA + 250 ml hypotonic beverage I: 50 mg GABA + 250 ml hypotonic beverage C: 250 ml hypotonic beverage	Single dose^*^	Single-blind, controlled, crossover	POMS, cortisol, CgA	Pre-ingestion, at the midpoint of the task (15 min post-ingestion), and after the completion of the task (35 min post-ingestion)
Nakamura et al. (2009)	*Experiment 1* 12 (males only, age range: 30–41, healthy) *Experiment 2* 12 (males only, mean age: 31–42, healthy)	*Experiment 1* I: 28 mg GABA in 10 g chocolate C: 20 g chocolate *Experiment 2* I: 28 mg GABA in 10 g chocolate C: 20 g chocolate	*Experiment 1* Single dose^*^ Experiment 2 Single dose^*^	*Experiment 1* Double-blind, controlled, crossover *Experiment 2* Double-blind, controlled, crossover	*Experiment 1* HRV (HF, LF, HFnu, LF/HF) *Experiment 2* CgA	Pre-ingestion, at the midpoint of the task (30 min post-ingestion), after the completion of the task (50 min post-ingestion)
Okita et al. (2009)	7 (males only, age range: 21–24, healthy)	I: 31.8 mg GABA in a vegetable tablet C: Placebo tablet with Dextrin + Potato starch + Gardenia	Single dose	Double-blind, controlled, crossover	HR, HRV (LF, HF, LF/HF), BP (SBP, DBP, MBP)	30 min-pre ingestion, 20, 40, and 60 min post-ingestion
Yamatsu et al. (2016)	10 (6 males, age range: 24–57, poor sleepers: PSQI >6)	I: 100 mg GABA + 4.7 mg Glutamic acid, 2.3 mg other amino acids, 3.4 mg minerals and 1.6 mg water (112 mg)—capsule C: 112 mg dextrin	1 week	Single-blind, controlled, crossover	PSQI Total, VAS for sleep satisfaction, easiness to fall asleep, and feelings upon awakening, EEG for sleep latency, sleep efficiency, Non-REM sleep latency, REM sleep time, Non-REM sleep time, Light Non-REM sleep time, Deep Non-REM sleep time, Awakening frequency, Delta wave	Pre-ingestion and post-ingestion, exact timelines are not clear
Yamatsu et al. (2015)	16 (7 males, age range: 27–45, poor sleepers: PSQI >6)	I: 100 mg GABA + 50 mg dextrin (capsule) I: 100 mg GABA + 50 mg AVLE (capsule) I: 50 mg AVLE + 100 mg dextrin (capsule) C: 150 mg dextrin (capsule)	1 week	Single-blind, controlled, crossover	PSQI Total, VAS for sleep satisfaction, easiness to fall asleep, and feelings upon awakening, EEG for sleep latency, Non-REM sleep latency, REM sleep time, Non-REM sleep time, Awakening frequency, Delta wave	Pre-ingestion and post-ingestion, exact timelines are not clear
Yoto et al. (2012)	63 (28 males, age range: 20–28, healthy)	I: 100 mg GABA (capsule) C: 100 mg dextrin (capsule)	Single dose^*^	Single-blind, controlled, crossover	POMS-tension/anxiety, VAS for arousal and relaxation, EEG-alpha and beta wave	Pre-ingestion, 10, 40, and 70 min post-ingestion
Abdou et al. (2006)	*Experiment 1* 13 (7 males, age range: 21–35, healthy) *Experiment 2* 8 (5 males, age range: 25–30, acrophobic)	*Experiment 1* I: 100 mg GABA + 200 ml water I: 200 mg theanine + 200 ml water C: 200 ml water *Experiment 2* I: 100 mg GABA + 200 ml water C: 200 ml water	*Experiment 1* Single dose *Experiment 2* Single dose^*^	*Experiment 1* Controlled, crossover *Experiment 2* Controlled, parallel	*Experiment 1* EEG for alpha and beta wave *Experiment 2* IgA	*Experiment 1* Pre-ingestion, at the time of ingestion, 30 and 60 min post-ingestion *Experiment 2* Before crossing, at middle, and at the end of the bridge
Byun et al. (2018)	40 (10 males, age range: 30–64, poor sleepers: PSQI >5 and ISI >8)	I: 300 mg GABA + maltodextrin (tablet) C: Maltodextrin (tablet)	4 weeks	Double-blind, controlled, parallel	ISI, PSQI for total score, sleep quality, sleep latency, total sleep time, sleep efficiency, PSG for total sleep time, N1, N2, N3, REM, WASO, Arousal Index, AHI, RDI, REM sleep latency, sleep latency, sleep efficiency	Pre-intervention and at the end of Week 4
Yamatsu et al. (2013)	38 (14 males, age range: 71–92, healthy)	I: 100 mg GABA in 6.8 g chocolate C: Dextrin in 6.8 g chocolate	4 weeks	Double-blind, controlled, parallel	Cortisol, OSA sleep inventory	Pre-ingestion, 30 and 60 min after ingestion

* *Utilized a stress task; HRV, heart rate variability; TP, total power; LF, low frequency power; HF, high frequency power; LF/HF, low frequency/high frequency ratio, HFnu: normalized high frequency power; VAS, visual analog scale; ACTH, adrenocorticotropic hormone; CgA, chromogranin A; POMS, profile of mood states; HR, heart rate; BP, blood pressure; PSQI, Pittsburgh sleep quality index; EEG, electroencephalography; REM, rapid eye movement; IgA, immunoglobulin A; ISI, insomnia severity index; PSG, polysomnography; WASO, wake after sleep onset; AHI, apnoea-hypopnea index; RDI, respiratory distress index; OSA, Oguri-Shirakawa-Azumi*.

**Table 2:** Summary of the studies—outcomes.

**References**	**Stress effects**	**Sleep effects**
Hinton et al. (2019)	- In both stress groups (low and high) both teas (regular oolong and GABA oolong) tea increased average RR intervals. However, GABA oolong (vs. regular oolong) had a greater significant influence—bigger increase on HRV (a change in in the RR interval) in high (vs. low) stressed individuals. - Immediate stress questionnaire, TP, LF, HF, LF/HF—not significant	- Not measured
Yoshida et al. (2015)	- Improved calmness and worry scores in GABA (vs. control) group at the 4th week of treatment - Trends for reduced cortisol and increased adiponectin in GABA (vs. control) group at the 8th (final) week of treatment - ACTH: not significant	- A trend for improved feelings of awakening in GABA (vs. control) group at the 4th and 10th weeks of treatment - VAS sleepiness score- not significant
Okada et al. (2000)	- Not measured	- Improved rate for sleep disturbance scores (derived from Kupperman Index) in GABA (vs. control) condition after 4 weeks of use−68% of improvement
Fujibayashi et al. (2008)	- Increased TP in GABA condition 30 and 60 min after ingestion (vs. baseline) - Increased HF in GABA condition 30 min after ingestion (vs. baseline) - TP and HF: no difference between GABA and control conditions - LF: not significant	- Not measured
Yamatsu et al. (2015)	- Reduced CgA in GABA coffee (vs. water and coffee) condition 30 min after administration	- Not measured
Kanehira et al. (2011)	- Lower cortisol and CgA after ingestion of both 25 and 50 mg GABA (vs. control) only in chronic fatigue group - Tension/anxiety score—not significant	- Not measured
Nakamura et al. (2009)	*Experiment 1* - Lower LF/HF and higher HF power values in GABA (vs. control) condition 6.5–9.5 min after the task (= 36.5–39.5 min after ingestion) - Higher HF power values in GABA (vs. control) condition 12–15 min after the task (= 42–45 min after ingestion) *Experiment 2* - Higher CgA values (due to stress task) in placebo condition 30 and 50 min (vs. baseline) after ingestion. Not observed in GABA condition	- Not measured
Okita et al. (2009)	- Increased LF/HF ratio and HR in control condition 20 and 40 min after intake but this increase was not observed for GABA condition - Stroke volume, cardiac output, HF and LF power, SBP, DBP, MBP—not significant	- Not measured
Yamatsu et al. (2016)	- Not measured	- Improved feelings upon awakening score, reduced sleep latency and increased total Non-REM (N1, N2, N3) sleep time in GABA (vs. control) condition after treatment - Trends for improved PSQI, sleep satisfaction, and ease of falling asleep scores and increased light Non-REM (N1, N2) sleep time and sleep efficiency in GABA (vs. control) condition after treatment - Deep Non-REM (N3-SWS), sleep latency and time, REM sleep time, awakening frequency, and delta wave power—not significant
Yamatsu et al. (2015)	- Not measured	- A trend for reduced sleep latency in GABA (vs. control) condition after treatment - PSQI total, sleep satisfaction, feeling of awakening, ease of falling asleep, deep Non-REM (N3-SWS) sleep latency, REM sleep time, Non-REM sleep time, awakening frequency and delta wave power—not significant
Yoto et al. (2012)	- Alpha and beta waves decreased from 20 to 60 min after intake (due to stress task) but 30 min after GABA intake, this decrease diminished in GABA (vs. control) condition - Tension/anxiety, arousal, and relaxation scores—not significant	- Not measured
Abdou et al. (2006)	*Experiment 1* - Increased changes for alpha waves (%) in GABA (vs. placebo) condition - Increased changes for alpha/beta ratio (%) in GABA (vs. placebo and theanine) condition - A trend for reduced changes for beta waves (%) in GABA vs. placebo condition *Experiment 2* Decreased IgA in control (vs. GABA) group at the middle and end of the bridge	- Not measured
Byun et al. (2018)	- Not measured	- Reduced sleep latency in GABA (vs. control) group after treatment - Decreased ISI, PSQI total, PSQI-sleep quality and PSQI-total sleep time scores in GABA group (pre vs. post-treatment)—but no between group differences - PSQI-sleep latency, PSQI-sleep efficiency, N1(%), N2(%), N3(%), REM (%), WASO(min), REM-sleep latency, arousal index, AHI, RDI—not significant
Yamatsu et al. (2013)	- Increased cortisol in placebo group after 2 and 4 weeks but not in GABA group	- Improved onset and maintenance of sleep, drowsiness in the morning, and recovering from fatigue scores in GABA group after 4 weeks but no placebo vs. GABA group analyses done

*HRV, heart rate variability; ACTH, adrenocorticotropic hormone; HF, high frequency; LF, low frequency; CgA, chromogranin A; SBP, systolic blood pressure; DBP, diastolic blood pressure; MBP, mean blood pressure; REM, rapid eye movement; PSQI, Pittsburgh sleep quality index; ISI, insomnia severity index; N1, Stage 1 Non-REM sleep; N2, Stage 2 Non-REM sleep; N3-Stage 3 Non-REM sleep; WASO, wake after sleep onset; AHI, apnoea-hypopnea index; RDI, respiratory distress index*.

### The Effect of GABA Consumption on Stress

Two experimental studies examined the effect of consuming GABA-enriched natural products (such as tea and rice) on stress. Although the majority of participant groups were reported to be healthy, the dose of GABA, duration of the intervention, and measures used to assess stress varied considerably.

A recent study by Hinton et al. (2019) investigating the acute effects of GABA Oolong consumption on stress demonstrated that in both (low and high) stress groups, both GABA Oolong tea (2.01 mg GABA/200 ml tea) and standard Oolong tea (0.25 mg GABA/200 ml tea) increased average RR interval (the time between two consecutive R waves in the electrocardiogram). However, GABA Oolong had a greater influence on heart rate variability (HRV), eliciting a bigger change in RR interval in high compared to low stressed individuals. The effects of GABA on other HRV parameters and subjective stress were not significant. Another study by Yoshida et al. (2015) showed that 8 weeks consumption of GABA rice (16.8 mg GABA in 150 g GABA rice/day) improved subjective calmness and worry scores midway through the study at the 4th week of the treatment compared to white rice (4.1 mg GABA in 150 g GABA rice/day), however, these effects were not maintained. They also reported trends for reduced blood cortisol and increased adiponectin levels in GABA rice (vs. white rice) condition at the 8th week of the treatment. However, they did not observe any effects on adrenocorticotropic hormone (ACTH) at any stage of the intervention.

All of the eight experimental studies investigated the effects of (i) single (Abdou et al., 2006; Fujibayashi et al., 2008; Nakamura et al., 2009; Okita et al., 2009; Kanehira et al., 2011; Yoto et al., 2012; Yamastsu et al., 2015) and (ii) repeated (Yamatsu et al., 2013) biosynthetic GABA consumption on stress—mainly on psychophysiological parameters, with doses ranging between 20-−100 mg and participant numbers between 7–63.

Fujibayashi et al. (2008) showed that 30 mg GABA ingestion increased (i) total power (TP) 30 and 60 min after ingestion compared to baseline and (ii) high frequency power (HF) 30 min after ingestion compared to baseline, however they failed to show between group differences and differences in other HRV parameters. In contrast, Okita et al. (2009) reported that the placebo tablet increased the LF/HF ratio and heart rate (HR) 20 and 40 min after consumption. This increase was not observed in the GABA condition (31.8 mg GABA), but they did find effects of GABA consumption on other parameters including stroke volume, cardiac output, HF and LF power, systolic blood pressure (SBP), diastolic blood pressure (DBP), and mean blood pressure (MBP). Using electroencephalography (EEG), experiment 1 from Abdou et al. (2006) showed that 100 mg GABA in 200 ml water increased changes in (i) alpha waves (compared to water condition) and (ii) alpha/beta ratio (compared to water and theanine condition). They also reported a trend for reduced changes for beta waves in GABA vs. water condition. The only 4 week-long interventional study utilizing biosynthetic GABA observed an increase in cortisol levels in the placebo group after 2 and 4 weeks of GABA use, but GABA group did not show such an increase (Yamatsu et al., 2013).

The other studies utilized various methodologies to induce stress on participants. Yamastsu et al. (2015) utilized the Uchida-Kraepelin Psychodiagnostic Test (UKT; Kuraishi, 2000), an arithmetic task to induce stress, and demonstrated that 20 mg GABA in coffee (compared to coffee only and water conditions) reduced chromogranin A (CgA) levels 30 min after consumption. A similar study utilizing UKT and CgA levels (Kanehira et al., 2011) showed that consumption of both 25 and 50 mg GABA in a 250 ml hypotonic beverage (compared to hypotonic beverage only condition) lowered salivary CgA and cortisol in individuals with chronic fatigue. However, the subjective tension/anxiety score was not significant. Nakamura et al. (2009) measured both CgA and HRV and found that 28 mg GABA in 10 g chocolate, compared to 20 g chocolate alone, decreased the LF/HF power 6.5–9.5 min after the arithmetic task (i.e., = 36.5–39.5 min after ingestion) and increased the HF power 12–15 min after the arithmetic task (i.e., = 42–45 min after ingestion). They also reported that the CgA values increased in the chocolate only condition 30 and 50 min (vs. baseline) after ingestion, an effect not observed in GABA chocolate condition. The electrophysiological study by Yoto et al. (2012) employed EEG to demonstrate that the UKT decreased alpha and beta band power, whereas 30 min after a 100 mg GABA capsule (vs. placebo capsule) intake, this decrease had diminished. Although participants failed to report a subjective increase in relaxation and decrease in tension/anxiety and arousal scores. Unlike the studies above, experiment 2 from Abdou et al. (2006) on the other hand, utilized a real life stress task, where acrophobic participants were asked to cross a suspended bridge. They discovered that control groups immunoglobulin A (IgA) levels decreased at the middle and end of the bridge, but 100 mg GABA capsule groups IgA levels did not show this pattern.

### The Effect of GABA Consumption on Sleep

Two 8-week intervention studies examined the effect of consuming GABA-enriched rice on sleep in healthy individuals. Yoshida et al. (2015), studying healthy middle aged individuals with poor sleep, found a trend for improved feelings upon awakening in GABA rice (16.8 mg GABA in 150 g GABA rice/day) (vs. white rice−4.1 mg GABA in 150 g white rice/day) group at the 4th week of intervention and after 2 weeks of the intervention (i.e., at the 10th week). They did not find an effect of GABA rice on VAS sleepiness score. Conversely Okada et al. (2000) reported, in post-menopausal women, that consumption of 26.4 mg GABA rice 3 times a day (compared to control rice) improved insomnia score of Kupperman Menopause Index at the 4th week of the treatment. Additionally, only one 4-week long study examined the effect of biosynthetic GABA consumption on sleep in healthy elderly participants. Using the OSA sleep inventory they showed improvements in the onset and maintenance of sleep, drowsiness in the morning, and recovering from fatigue scores in the GABA group after 4 weeks of treatment, although they did not find differences between GABA and placebo groups (Yamatsu et al., 2013).

Three 1 to 3-week long intervention studies (albeit with very low sample sizes), investigated the effects of biosynthetic GABA consumption on sleep in individuals with poor sleep quality (one with PSQI > 5 scorers, and two with PSQI > 6 scorers; PSQI: Pittsburgh Sleep Quality Index). In their first 1 week long intervention study, Yamatsu et al. (2016) showed that the intake of 100 mg GABA capsule (vs. control) improved feelings upon awakening scores, objectively measured reduced sleep latency, and increased total Non-REM (N1, N2, and N3/SWS) sleep time after intervention. They also observed trends for improved PSQI, sleep satisfaction, and ease of falling asleep scores and increased light Non-REM sleep time and sleep efficiency in GABA (vs. control) condition after treatment. However, they did not find significant effects for deep Non-REM (N3/SWS) sleep latency and time (i.e., duration), REM sleep time, awakening frequency, or delta wave power. In their other 1 week intervention study, Yamatsu et al. (2015), studying middle aged sleepers who reported having poor sleep, observed a trend for reduced sleep latency only in 100 mg GABA capsule (vs. control) condition. Results from PSQI total, sleep satisfaction, feeling of awakening, ease of falling asleep scores and Non-REM sleep latency, REM sleep time, Non-REM sleep time, awakening frequency and delta wave power were not significant for GABA only vs. other intervention (AVLE and AVLE+GABA) and control groups. The most recent 4 week long intervention study in this area by Byun et al. (2018), studying middle aged sleepers who reported having poor sleep, reported that 300 mg GABA tablet (vs. control tablet) intake reduced sleep latency after the intervention. They also found that N2 sleep (%) and insomnia severity index (ISI) decreased, as did PSQI total, PSQI-sleep quality, PSQI-sleep latency and PSQI-total sleep time scores in GABA group (pre vs. post-treatment), however, they failed to find GABA vs. placebo/group differences. Additionally, there were no statistically significant effects of PSQI-sleep efficiency scores, and total sleep time, stage 1, and 3 Non-REM sleep (%), REM (%), wake after sleep onset (WASO; min), REM-sleep latency, sleep efficacy, arousal index, apnoea-hypopnea index (AHI), and respiratory distress index (RDI).

## Discussion

### Summary of the Main Results

This systematic review aimed to establish the current status of knowledge regarding the effects of natural and biosynthetic GABA consumption on stress and sleep. Overall, our review of the literature showed that there was low to moderate evidence for GABA's stress (due to the fact that there are more studies with positive results) and low evidence for GABA's sleep benefits.

Methodologies of the studies included in this review varied significantly but included both subjective and objective measures of stress and sleep. The majority of the studies did not find significant subjective improvements of stress scores after consuming a single dose of either natural or biosynthetic forms of GABA. Evidence for extended GABA use is mixed, Yoshida et al. (2015) study which reported improved calmness and worry scores in GABA (vs. control) group at the 4th week of treatment, but not with GABA use beyond that. On the other hand, only some of the subjective sleep scores including sleep disturbance, feelings upon awakening, onset and maintenance of sleep, drowsiness in the morning, and recovery from fatigue scores improved only when there was a prolonged GABA use for at least 1 week (Okada et al., 2000; Yamatsu et al., 2013, 2016). Remaining studies showed either trends toward improvements or insignificant subjective improvement of sleep. It may well be the case that prolonged natural GABA use is required to elicit subjective stress and sleep benefits.

### Stress

Due to GABA's BBB permeability issues, most of the studies utilized autonomic nervous system (ANS)-related measures (such as HRV, cortisol, and CgA) to examine the impact of GABA consumption on stress. Abdou et al. (2006) and Yoto et al. (2012) utilized EEG to evaluate the central action of GABA. The studies using ANS-related measures showed positive but rather conflicting results. Hinton et al. (2019) reported increased RR intervals in the GABA condition that reflects more stable ANS function through an increase in vagal activity (indicative of reduced stress response) (Camm et al., 1996). Similarly, although no treatment differences were reported between GABA and control conditions, Fujibayashi et al. (2008) showed an increased TP in GABA condition 30 and 60 min after ingestion (vs. baseline) which is indicative of ANS functionality and adaptability and reduced stress (Camm et al., 1996). The same study reported increased HF in GABA condition 30 min after ingestion (vs. baseline) which is indicative of increased PNS activity and reduced stress (Berntson et al., 1997). Increased RR intervals, TP and HF suggests that GABA exerts its effects by parasympathetic augmentation with no or smaller sympathetic effects.

The remainder of the studies that utilized ANS-measures showed the opposite activation pattern. LF/HF, a marker of SNS activity and sympathovagal balance which increases under stress conditions (Pagani et al., 1991) was either not increased (Okita et al., 2009) or reduced in the GABA condition (Nakamura et al., 2009). Similarly, CgA, a protein co-released with noradrenaline in the SNS (Dimsdale et al., 1992), and cortisol, a glucocorticoid hormone that is released by the adrenal cortex via (i) release of adrenocorticotropic hormone by regulation of hypothalamic–pituitary–adrenal axis and (ii) the SNS innervation (Engeland and Arnhold, 2005), were reduced in GABA vs. control conditions (Nakamura et al., 2009; Kanehira et al., 2011; Yamastsu et al., 2015), indicative of reduced stress levels. Also, IgA, a glycoprotein that is regulated by the SNS (Carpenter et al., 1998) that is lower in anxiety (Graham et al., 1988), was found to be decreased in control but not in GABA conditions during a stressful task (Abdou et al., 2006), suggesting a stress-protective effect of GABA. According to these studies, GABA induced relaxation by modulating the sympathetic nervous system.

Although there is no consensus regarding which division of the autonomic nervous system is most affected by GABA intake, there is limited evidence that GABA also crosses the BBB and exerts biological effects on the CNS. Stress reduction and relaxation are associated with enhanced alpha oscillations (Nobre et al., 2008), reduced beta activity (Ray and Cole, 1985), and increased alpha/beta ratio (Liang et al., 2019; Yi Wen and Mohd Aris, 2020). In line with this, Abdou et al. (2006) observed increased changes for alpha waves and alpha/beta ratio in GABA (vs. placebo) condition, suggesting improved relaxation. Similarly, Yoto et al. (2012) reported that both alpha and beta waves decreased due to a stress task, but 30 min after GABA intake, this decrease diminished in GABA (vs. control) condition, indicating a stress-protective effect of GABA. These results suggest that, GABA passes the BBB either in small or full amounts to exert biological effects on the CNS.

In summary, stress markers of both divisions of the ANS and the CNS seem to be affected by oral GABA intake. However, it is important to note that the efficacious doses for stress reduction and/or stress-protective benefits range from 2.01 to 100 mg, where the lower doses up to 30 mg seem to affect the autonomic markers of stress and a dose of 100 mg seems to affect the central markers of stress. Additionally, efficacious doses for natural GABA seem to be lower than that of the biosynthetic forms. Although natural GABA intake and stress research is very limited, these results may also be attributable to the other bioactive compounds found naturally in foods that have stress reduction benefits such as l-theanine (Juneja et al., 1999) and epigallocatechin gallate (EGCG) in tea (Scholey et al., 2012). Further studies are warranted to examine (i) natural and biosynthetic GABA bioavailability in humans following oral intake in order to understand GABA's mechanism of action for each type of GABA, (ii) the minimum and optimum natural and biosynthetic GABA doses required for stress benefits, and (iii) the minimum and optimum natural and biosynthetic GABA doses required to affect stress reduction/relaxation peripherally and centrally.

### Sleep

There is only very limited supportive evidence regarding the role of oral GABA intake on objective sleep improvement. Byun et al. (2018), whose participants were dosed 1 h before sleeping, reported that 4 weeks use of GABA reduced sleep latency in GABA (vs. control) group. Similarly, Yamatsu et al. (2016), with dosing 30 min before sleep, showed that 1 week GABA intervention reduced sleep latency and increased total Non-REM sleep time in GABA (vs. control) condition. However, in a previous study with the same dosing regimen, Yamatsu et al. (2015) only observed a trend toward reduced sleep latency after 1 week of GABA consumption. All three studies failed to show beneficial effects of GABA intake on other markers of sleep such as sleep efficiency, REM sleep time, awakening frequency etc. These findings suggest that prolonged GABA intake (i.e., repeated dosing across days) may be beneficial for naturally inducing sleep rather than maintaining sleep, as evidence showed that GABA primarily affects sleep onset and early stages of sleep that occur early at night (i.e., the first Non-REM of the night), but not the stages of sleep that occur later at night. This could be explained by the pharmacokinetic profile of GABA, characterized by a rapid increase (30 min after oral administration) and then decrease (60 min after oral administration) in plasma concentrations. In other words, the quick elevation in the blood GABA levels might explain as to why it differentially affects early sleep markers. Additionally, there is a bi-directional relationship between sleep and both acute and chronic anxiety where sleep disturbance is observed in individuals with anxiety (Soehner and Harvey, 2012) and having a sleep disturbance may predict the development of an anxiety disorder (Neckelmann et al., 2007). Specifically, increased sleep onset latency has been observed in anxiety and related disorders (Cox and Olatunji, 2016) and stress (Maskevich et al., 2020). Therefore, early sleep stage-related benefits of GABA consumption could be associated with GABA's stress reduction properties, rather than direct sleep inducing and/or maintaining benefits *per se*. The lack of sleep maintenance-related benefits of GABA might also be explained by (i) small and unequal group sizes that mask real improvements, (ii) insufficient GABA amounts that does not drive SWS and REM responses, and (iii) not utilizing split-night PSG/EEG and masking the significant changes that may only be evident in different parts of the night.

Repeated GABA intake across days may improve early sleep parameters; however, it is important to note that doses required to elicit sleep benefits (ranging between 100 and 300 mg for biosynthetic GABA) seem to be higher than that of stress benefits (ranging between 20 and 100 mg for biosynthetic and 2.01 and 26.4 mg for natural GABA) and seem to require a long-term use (1–8 weeks) to improve early sleep measures only. Having said that, doses ranging between 100 and 300 mg seems to be efficacious in reducing sleep latency with prolonged use of 1–4 weeks. Again, it is important to note that all sleep studies which reported improved objective sleep measures utilized biosynthetic forms of GABA. Future research is required to understand (i) the minimum and optimum natural and biosynthetic GABA doses required to affect different stages of sleep, and (ii) whether lower doses might be more efficacious for peripheral markers of sleep.

### Limitations

The current review was subject to several limitations. Firstly, the quality of many of the reviewed studies was questionable due to potential conflicts of interest, low participant numbers, and unequal control and intervention groups. Secondly, not all studies have assessed the same stress and/or sleep parameters or employed the same design, hence no quantitative meta-analysis could be performed due to heterogeneity of the extracted data. Thirdly, although PICOS (patient-intervention-control-outcome-study design) was used to extract data, there was only one data extractor and no validated tool has been used. Finally, due to the limited number of heterogenous studies in this area, the precise dose for efficiency for both stress and sleep benefits could not be established neither in the current review nor in the general scientific literature. Hence, the current review encourages future studies to examine dose-response relationships between oral natural and biosynthetic GABA consumption and stress and sleep by using self-report, behavioral, peripheral, and neurophysiological markers of stress and sleep.

## Conclusion

This review offers a comprehensive assessment of the current GABA literature and shows that natural and biosynthetic GABA intake may have beneficial effects on stress and sleep. However, due to small sample sizes and heterogeneity of methods used, further research is warranted to establish dose timing, duration, and response relationships for both natural and biosynthetic forms of GABA to reliably elicit acute or chronic stress and sleep effects.

## Data Availability Statement

All datasets presented in this study are included in the article.

## Author Contributions

PH wrote the manuscript with input from JG, JN, and AS who also contributed to the revision of the manuscript critically for important intellectual content. All authors contributed to the article and approved the submitted version.

## Conflict of Interest

PH is employed by Unilever UK Central Resources Limited. JG, JN, and AS have received research funding, consultancy, travel support, and speaking fees from various industrial companies.
